# The Potential for Lifestyle Intervention Among Patients Undergoing Transurethral Resection of Bladder Tumour Based on Patient Needs Including Smoking and Other Risky Lifestyle Factors: A Cross-Sectional Study

**DOI:** 10.3390/ijerph21121633

**Published:** 2024-12-08

**Authors:** Line Noes Lydom, Susanne Vahr Lauridsen, Ulla Nordström Joensen, Hanne Tønnesen

**Affiliations:** 1 WHO-CC/Clinical Health Promotion Centre, The Parker Institute, Bispebjerg and Frederiksberg Hospital, University of Copenhagen, 2000 Frederiksberg, Denmark; 2 Centre for Perioperative Optimization, Department of Surgery, Herlev Hospital, University of Copenhagen, 2730 Herlev, Denmark; 3 Department of Clinical Medicine, University of Copenhagen, 2200 Copenhagen, Denmark; 4 Department of Urology, Copenhagen University Hospital Rigshospitalet, University of Copenhagen, 2100 Copenhagen, Denmark

**Keywords:** transurethral resection of bladder tumours, NMIBC, prehabilitation, smoking cessation, surgery

## Abstract

Bladder cancer is the tenth most common cancer worldwide, with non-muscle invasive bladder cancer (NMIBC) accounting for 75% of cases. Transurethral resection of bladder tumours (TURBT) is the standard treatment, but it is associated with significant risks of complications and recurrence. Risky lifestyle factors, including smoking, malnutrition, obesity, risky alcohol use, and physical inactivity (collectively termed SNAP factors), may worsen surgical outcomes and increase cancer recurrence. Prehabilitation programmes targeting these modifiable risk factors could improve patient outcomes. This cross-sectional study assessed 100 TURBT patients at a Danish university hospital to determine the prevalence of SNAP factors and the potential for lifestyle interventions. Data were collected via structured interviews, and intervention scenarios were projected based on efficacy rates of 5–100%. In total, 58% of patients had at least one risky SNAP factor, with smoking (29%) being the most prevalent, followed by physical inactivity (19%) and risky alcohol use (18%). Obesity (7%) and malnutrition (8%) were less common. Seventeen percent had multiple SNAP factors. No significant demographic indicators were associated with the presence of SNAP factors. TURBT patients with NMIBC show a high prevalence of risky lifestyle factors, including smoking and obesity, with over half affected. Systematic screening and targeted interventions could significantly improve patient outcomes and long-term health.

## 1. Introduction

Bladder cancer is the tenth most common cancer type worldwide, with approximately 573,000 new cases reported in 2020 [1,2]. Approximately 75% of these patients were diagnosed with non-muscle invasive bladder cancer (NMIBC) [2], for which transurethral resection of the bladder tumour (TURBT) remains the gold standard for both diagnosis and treatment [3]. The treatment of NMIBC typically requires multiple follow-ups in the healthcare system in the years following diagnosis, including procedures such as Bacillus Calmette-Guerin (BCG) or chemotherapy instillations, regular follow-up cystoscopies and TURBT to control and treat recurrences. As a result, many NMIBC patients are in a cycle between the preoperative and the postoperative phases. Treatments for NMIBC consume substantial resources for both patients and society [4]. TURBT is a procedure usually performed under general anesthesia, requiring hospital admission, and is associated with significant lower urinary tract symptoms in the days or weeks following the procedure. The procedure has a complication rate ranging from 11% to 23%, with haematuria and urinary tract infection being the most common [5,6]. It is well established that even with a low risk of high-grade complications for individual TURBT procedures, the burden of the disease, repeated transurethral procedures, and surveillance have a negative impact on quality of life (QoL), including decreased physical function and mental health [7].

Smoking is a well-established risk factor for the development of bladder cancer [8]. Smokers face a significantly elevated risk of recurrence following treatment and smoking may decrease the efficacy of adjuvant intravesical BCG treatment after TURBT [9]. Specifically, current smokers had a higher risk of recurrence than former smokers, with an odds ratio (OR) of 1.24 (95% CI: 1.02–1.50) [10]. A systematic review suggests that overweight and obesity were also linked to a higher risk of recurrence in patients with NMIBC, with hazard ratios (HRs) of 1.29 (1.05–1.58) and 1.82 (1.12–2.95), respectively [11]. Thus, there seems to be considerable potential for intervening to improve risk of recurrence and reduce negative impact of multiple treatments on QoL during the disease and treatment course that often lasts for years in NMIBC patients.

Interestingly, the same risk factors are also important when patients undergo surgery. Overall, smoking during the time of surgery increases the risk of postoperative complications; the relative risk (RR) is 1.52 (1.33–1.74) [12], and obesity has been associated especially with infectious complications [13,14,15]. Other risky lifestyle factors, including malnutrition, excessive alcohol use, and physical inactivity, also impact surgical outcomes [16,17,18].

Today, the patients undergoing TURBT should be offered smoking cessation intervention. In the future, they may also be candidates for obesity intervention based on further research regarding the impact of obesity on recurrence. In case of progression to muscle invasive bladder cancer, they are candidates for the whole prehabilitation programme, including smoking, obesity, malnutrition, excessive alcohol use, and physical inactivity (collectively termed SNAP) prior to cystectomy [19,20], which, even in the age of enhanced recovery after surgery, still carries a high risk of serious complications and loss of function after surgery [21]. The NMIBC stage may therefore additionally be a window of opportunity for lifestyle changes that can benefit patients in the event that there will later be a need for extensive cancer surgery with limited time for such interventions.

Although interest in the burden of several lifestyle factors in the TURBT population has increased over recent years [22,23,24,25], until now, no studies have mapped smoking and the other SNAP factors in this population.

Therefore, this study aimed to identify the prevalence of smoking and other SNAP factors in patients undergoing TURBT and estimate the potential for lifestyle change. Additionally, we identified indicators for the group that required lifestyle interventions. We hypothesized that the TURBT population has high potential for smoking cessation and other lifestyle interventions.

## 2. Materials and Methods

### 2.1. Study Design, Participants, and Setting

**Figure 1:**
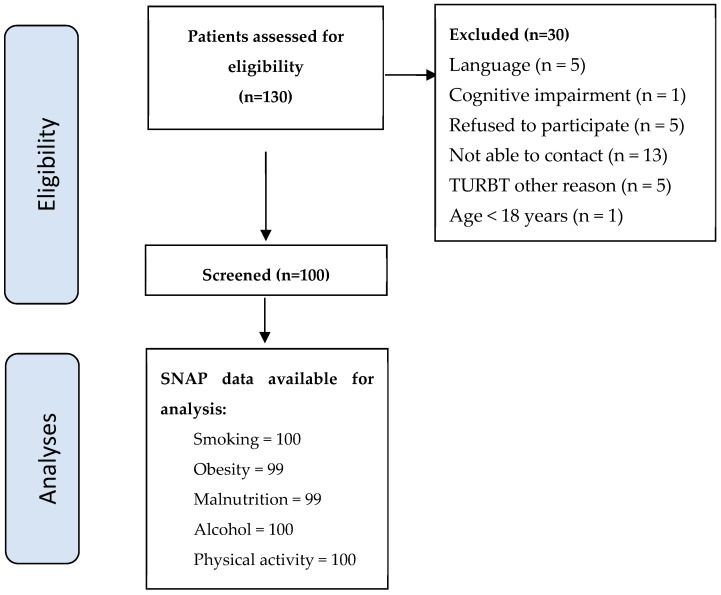
Patient flow. Abbreviations: SNAP: smoking, obesity, malnutrition, alcohol, and physical inactivity (collectively termed SNAP).

This was a cross-sectional study involving patients undergoing TURBT at a Danish university hospital. A total of 130 adult patients (18 years of age or older) scheduled for TURBT were assessed for eligibility. Of these, 30 patients were excluded for various reasons (see Figure 1), leaving 100 patients in this study (see Table 1 for demographic characteristics and lifestyle data). Since the documentation of risky SNAP factors is part of a surgical journal, data were systematically collected and prospectively recorded in the electronic medical records by a clinical trial coordinator nurse (from 9 November 2023, to March 2024) and imported into a REDCap database, an electronic data capture tool hosted in the Capital Region, Denmark [26]. Patients were excluded if they were unable to participate in the telephone interview due to language barriers or cognitive impairments, if contact could not be established after three attempts, if they declined participation, if the procedure was wrongly registered as a TURBT, or if the patients were previously screened. This study was approved by the Danish Scientific Ethical Committee (H-20081571) and the Danish Data Protection Agency (P-2020-95) as a part of the STRONG for surgery, Strong for life study, and reported in line with the STROBE statement [27].

### 2.2. Data Sources and Variables

Patient-reported data were collected via a structured telephone interview on height in centimetres, weight in kilograms, weight loss during the last three months, reduced food intake during the last week, alcohol intake exceeding two alcohol units per day or 14 units per week (one unit = 12 g of alcohol), physical activity less than 30 min per day, smoking status as current (daily smoking), former, or never, the total number of years they had smoked, and the average number of cigarettes smoked per day during those years. Additionally, age at the day of the interview, sex, living situation, and educational background were collected.

Body mass index (BMI) was calculated from body weight and height. The nutritional risk score was calculated from recent weight loss, reduced food intake, BMI, age, and data on the severity of disease from electronic medical records [28].

### 2.3. Outcome Assessments

The frequency for individual and coexisting SNAP factors were assessed. Secondly, need was estimated based on scenarios of interventional effects ranging from 5% to 100%. Finally, important indicators for having risky SNAP factors, such as age, sex, educational background, or living situation, were identified. Age, originally a continuous variable, was dichotomized into two groups: ≤68 years and >68 years. The cutoff of 68 years was selected based on the median age of the study population.

### 2.4. Statistical Methods

Patient inclusion was limited to an 18-week period when this study could be conducted at the surgical department.

Descriptive statistics were employed to summarize the potential benefits of lifestyle intervention and the effects of the intervention. Continuous variables are reported as the median with interquartile range (IQR), whereas categorical variables are presented as frequency and proportion. To identify key indicators related to age (≤68 years/>68 years), sex (female/male), education (higher than short or no education/short or no education), and living situation (not living alone/living alone), a comparison between patients with and without risky lifestyle factors was conducted via logistic regression. Both univariate and multivariate analyses were performed, with the results expressed as odds ratios (ORs) and 95% confidence intervals (CIs). An indicator was considered statistically significant if its confidence interval did not include the value 1. The data were analyzed with R statistical software version 4.1.0^®^.

## 3. Results

### 3.1. Frequency of SNAP Factors

**Figure 2:**
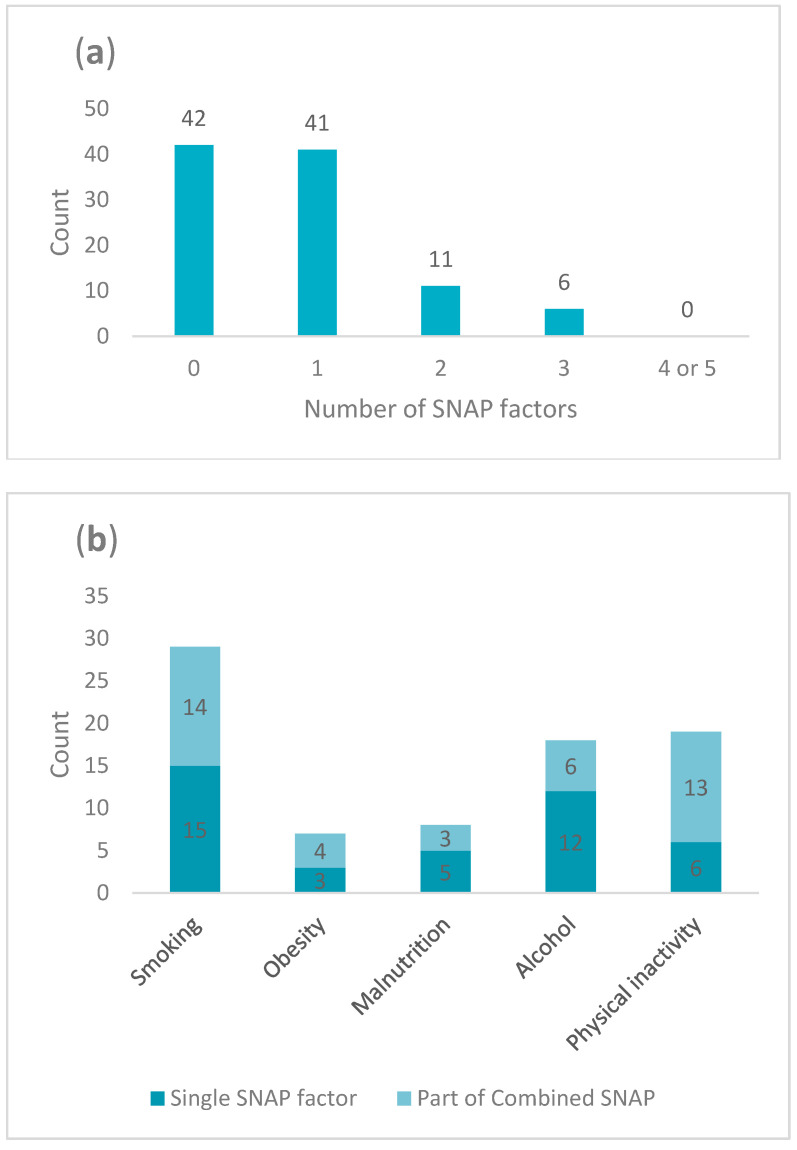
Frequency of risky SNAP (smoking, malnutrition, obesity, alcohol and low physical activity) factors; (**a**) represents the prevalence of 0–5 SNAP factors, (**b**) represents the distribution of the SNAP factors either individually (dark blue) or in combination of two or more SNAP factors (light blue), e.g., for tobacco, 15 patients were smokers exclusively and 14 were smokers in combination with one or more co-existing SNAP factors.

A total of 58% of patients had at least one risky lifestyle factor (see Table 1 and Figure 2a). Smoking was most common, with 29% of patients being daily smokers. The second most common risky SNAP factors were alcohol use and physical inactivity, affecting 18% and 19% of patients, respectively. The least prevalent risky SNAP factors included obesity and risk of malnutrition, impacting 7% and 8% of patients, respectively (see Figure 2b).

#### Coexistence of SNAP Factors

Seventeen percent of the patients exhibited coexisting risky SNAP factors, with 11% having two and 6% having three risky SNAP factors. The most common combination of risk factors was smoking and physical inactivity, which was observed in 4% of the population. Each of the following combinations affected 2 percent, i.e., smoking and alcohol and alcohol and physical inactivity, as well as combinations of three factors, i.e., smoking, obesity, and physical inactivity; smoking, alcohol, and physical inactivity; and smoking, malnutrition, and physical inactivity. The least prevalent combinations, each affecting 1% of patients, included obesity and physical inactivity, smoking and malnutrition, and smoking and obesity.

### 3.2. Hypothetical Scenarios of the Potential for Smoking Cessation and Other Lifestyle Changes

**Figure 3:**
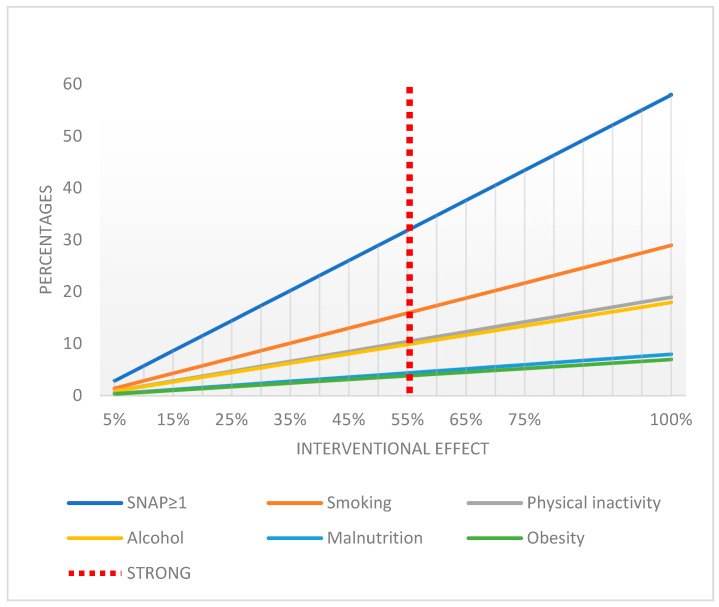
Hypothetical scenarios of potential results after smoking cessation and other SNAP interventions with different intervention effect scenarios for the study population.

Figure 3 presents scenarios of implementation of different interventions with varying efficacy rates ranging from 5% to 100% that could be offered. Based on these scenarios, a potential reduction in the prevalence of risky SNAP factors was projected. Hypothetically, for our study population, a smoking cessation intervention with 50% efficacy would mean that 14% to 15% would change from having a risky lifestyle to a healthier lifestyle; in comparison, an intervention targeting obesity with an efficacy of 50% would result in 3.5% of the total population changing to a healthier lifestyle.

These findings highlight the significant potential to improve the health outcomes of patients by targeting the most prevalent risk factors, particularly smoking, physical inactivity, and risky alcohol use.

### 3.3. Indicators for No Risky Lifestyle Factors

When patients with and without risky SNAP factors were compared, univariate analysis revealed no significant differences between the groups concerning age, sex, living alone, or a short-term or no education (see Table 2). Similarly, the multivariate analysis did not reveal any significant indicators; this suggests that, in this population, demographic characteristics such as age, sex, and educational background were not strong predictors of whether a patient exhibited risky lifestyle factors.

## 4. Discussion

Our study revealed that almost one in three patients were smokers and only one in fourteen were obese. Overall, more than half of the patients who underwent TURBT had one or more modifiable risky SNAP factor.

The prevalence of smokers in our study was notably higher than the 12% in the general adult population of Denmark [29]. This suggests a strong need for smoking cessation intervention for our patient group, which is in line with the European Association of Urology guidelines strongly recommending smoking cessation, as integrated in the treatment pathway of NMIBC [30]. The high frequency of smokers in the postdiagnostic period has been described previously [31].

It is a clinical experience that surgery is a window of opportunity for lifestyle changes. However, the results show a wide range of successful quitting due to the intensity of the cessation programme. Systematic reviews have shown that it is possible to successfully halve the number of patients with smoking in the perioperative period when using the longer-term intensive interventions [32,33]. In contrast, the briefer interventions including the motivational interviewing technique result in successful quitting in one in ten persons only [32,33]. It seems obvious to use the most effective intervention for the NMIBC patients who may benefit not only from short-term intervention at the time of the individual TURBT, but also from longer-term interventions for subsequent procedures or need for more extensive surgery later on.

More than a decade ago, a systematic review revealed that smoking cessation for more than ten years significantly reduced recurrence by one-third and halved progression among this patient group [34]. Recently, two other systematic reviews have been published, including 28 studies with 7885 patients and nine studies with 5490 patients [10,35]. Caini and co-workers followed patients postdiagnosis for a median of 3.2–6.7 years and reported no impact of quitting on recurrence or progression [35]. In contrast, Slusarczyk and co-workers reported that current smokers had a greater risk of recurrence than both former smokers and never smokers. In addition, former smokers had a greater risk than never smokers, thus indicating an effect of quitting. However, the follow-up period for recurrence only ranged from 3 to 72 months after diagnosis, while smoking history was likely longer and included a prediagnostic period [10].

In comparison with our prevalence of 29% smokers, other studies on this patient group from the United States, the Netherlands, and France reported smoking prevalence rates of 7% [24], 16% [25], 32% [22], respectively. As NMIBC is closely related to smoking, it seems that more smokers have successfully quit in the United States and the Netherlands than in France and Denmark. Learning from best practises across countries would be informative.

Obesity has been suggested as a risk factor for the recurrence of NMIBC [11]; however, we have not identified studies evaluating the effect of obesity intervention on weight reduction and recurrence in this patient group. In comparison with our prevalence of 7% patients being obese, other studies ranged from 18% to 19% [22,25] to 32% [24].

Likewise, we were not able to identify intervention studies on all risky SNAP factors or combinations of them. Notably, the STRONG programme with a proposed efficacy of 55% on both individual and coexisting risky SNAP factors could result in approximately one-third of our study population changing to a healthier lifestyle [36].

Neither univariate nor multivariate analyses identified significant differences between the group with and without risky SNAP factors regarding age, sex, living alone, or having a short or no education. Interestingly, the analysis suggested a tendency for living alone to be associated with lower odds of having one or more risky SNAP factors. This contrasts with findings from the general Danish population, where smoking but not obesity is more prevalent among individuals who are single and lowest among those who are married [37].

### 4.1. Strengths, Bias, and Limitations

The systematic and prospective collection of data based on structured telephone interviews, rather than relying on patients to fill out a questionnaire independently, was a strength [38].

While our study provides valuable insights, it is not without bias and limitations. Reliance on data reported at interviews for smoking and other risky SNAP factors may still introduce reporting bias, as patients may underreport or overreport their behaviours due to social desirability bias, thus overestimating healthy lifestyles. This study was conducted over a relatively short period (November 2023 to March 2024), which may not capture seasonal variations in risky SNAP factors, such as higher alcohol consumption and physical activity in summer period compared to winter [39,40]. The exclusion of those unable to be contacted for a phone interview may also have biased the results towards healthier participants.

When estimating the potential for lifestyle improvement through interventions, it is assumed that all patients would choose to participate in the offered intervention. However, interventional studies within the field of lifestyle intervention and prehabilitation for surgical populations have shown that up to 20–50% may decline participation [41,42,43,44].

The sample size was relatively small and therefore included a risk of type 2 failure. Larger cohorts may yield different results. This study was conducted at a single centre in a high-income country at a university hospital, which may increase the internal reliability but not the external reliability; this limits the generalizability of the findings to other hospitals, regions, low-income countries, rural areas, and cultures.

### 4.2. Perspectives

Patients have the possibility of reducing recurrence and disease progression through effective interventions, which may ease the burden of disease and treatment for both patients and their families. These interventions should be patient-centred and tailored to needs and preferences. Importantly, surgery or diagnosis offers a unique window of opportunity to engage patients when they may be more motivated to make lifestyle changes.

The clinical perspectives include training in systematic screening and the implementation of evidence-based cessation interventions for this patient group, which has a very high prevalence of smoking. When training healthcare providers in these interventions, is essential to ensure consistent and effective delivery of care.

From a management point of view, it is critical to integrate these programmes into routine care pathways, ensuring they are guided by evidence-based practises and aligned with both international guidelines and patient preferences to improve patient outcomes.

Future research should aim to include larger, more diverse populations, and consider a full-year timeline to capture seasonal variations as well as long-term follow-up. Lifestyle intervention at the time of diagnosis of NMIBC may further benefit those patients who will need multiple procedures or later progress to need more extensive cancer surgery such as cystectomy, where there is a larger potential to reduce the risk of serious complications and loss of function. In addition, it is important to disseminate best evidence-based practises to learn across countries.

## 5. Conclusions

This study revealed a high prevalence of smoking and other modifiable lifestyle factors among TURBT patients with NMIBC. Almost one in three were smokers and one in fourteen were obese, while over half of the population showed at least one risky SNAP factor. No indicators predicted these risks. Intensive intervention may halve the prevalence of patients with smoking and other risky SNAP factors.

Systematic screening for risky lifestyles and offering effective interventions are essential to improve the outcomes of the TURBT population in addition to the other long-term effects of lifestyle changes.

## Figures and Tables

**Table 1:** Patient characteristics. Presented as the number or median [IQR].

		N = 100
Patient characteristics		
Age in years		68 [59–76]
Male		80
Living alone		30
Education: short or none ^1^		23
Risky lifestyle factors		
Smoking:		
	Current	29
	Former	48
	Never	23
	Pack years ^2^	25 [13–46]
BMI ^1^		25.6 [23.8–27.8]
BMI < 18.5		1
BMI 18.5–24.9		43
BMI 25–29.9		48
BMI ≥ 30		7
NRS score ^1^		1 [0–2]
Malnutrition NRS ≥ 3		8
Alcohol consumption > 14 AU/week		18
Physical activity < 30 min/day		19
Number of risky lifestyle factors:		
	None	42
	One	41
	Two	11
	Three	6
	Four to five	0

Notes: Short or no education—only primary school or short work-related courses. Pack years = (number of cigarettes smoked per day) × (number of years smoked)/20. Abbreviations: AU: alcohol units, BMI: body mass index, NRS: nutritional risk score [28]. ^1^ Missing value for one patient; ^2^ missing data for one current and one former smoker.

**Table 2:** Characteristics of patients with no risky SNAP factors (N = 41) versus those with ≥1 risky SNAP factors (N = 58).

Patient Characteristics		No Risky SNAP Factors n (%)	≥1 risky SNAP Factors n (%)	OR [95%CI]	Adjusted OR [95%CI]
Age					
	Age > 68 years	18 (44)	27 (47)	1.11 [0.50–2.50]	1.24 [0.54–2.87]
Sex					
	Male	33 (80)	46 (79)	0.92 [0.33–2.50]	0.86 [0.29–2.44]
Living alone					
	Yes	15 (37)	15 (26)	0.61 [0.25–1.44]	0.53 [0.21–1.31]
Education					
	Short or none	7 (17)	15 (26)	1.69 [0.64–4.87]	1.89 [0.69–5.64]

Notes: One patient was censored from the analysis due to missing information regarding BMI. “Short or none” education refers to only primary school or short work-related courses. Abbreviations: CI: confidence interval, OR: odds ratio, SNAP: smoking, malnutrition, obesity, risky alcohol and low physical activity.

## Data Availability

The data presented in this study are available on request from the corresponding author due to restrictions (ethical reasons).
